# H5N1 Outbreaks and Enzootic Influenza

**DOI:** 10.3201/eid1201.051024

**Published:** 2006-01

**Authors:** Robert G. Webster, Malik Peiris, Honglin Chen, Yi Guan

**Affiliations:** * St. Jude Children's Research Hospital, Memphis, Tennessee, USA;; † University of Hong Kong, Hong Kong, SAR, China;; ‡ Shantou University Medical College, Shantou, Guangdong, China

**Keywords:** H5N1 influenza viruses, source, genesis, spread, ducks, migratory birds, vaccines, pandemic, perspective

## Abstract

Ongoing outbreaks of H5N1 avian influenza in migratory waterfowl, domestic poultry, and humans in Asia during the summer of 2005 present a continuing, protean pandemic threat. We review the zoonotic source of highly pathogenic H5N1 viruses and their genesis from their natural reservoirs. The acquisition of novel traits, including lethality to waterfowl, ferrets, felids, and humans, indicates an expanding host range. The natural selection of nonpathogenic viruses from heterogeneous subpopulations cocirculating in ducks contributes to the spread of H5N1 in Asia. Transmission of highly pathogenic H5N1 from domestic poultry back to migratory waterfowl in western China has increased the geographic spread. The spread of H5N1 and its likely reintroduction to domestic poultry increase the need for good agricultural vaccines. In fact, the root cause of the continuing H5N1 pandemic threat may be the way the pathogenicity of H5N1 viruses is masked by cocirculating influenza viruses or bad agricultural vaccines.

## Abstract 2

Highly pathogenic H5N1 influenza viruses continue to evolve and increase their geographic and host range.

Influenza is an ancient disease that has infected humans at irregular intervals throughout recorded history (*1*). While the 1918 "Spanish" influenza is the best recorded catastrophic influenza pandemic, similarly severe pandemics occurred earlier, when the human population of the world was much smaller, and they will occur again. Our challenge is to understand all aspects of the influenza virus, the hosts and their response, and the virus' global impact so that we may be better prepared to face the inevitable next influenza pandemic.

The influenza virus that appears most threatening is the avian H5N1 strain that since 2003 has infected >130 persons in Vietnam, Thailand, and Cambodia and has killed more than half of them. Nonetheless, the H5N1 influenza threat is viewed with disturbing complacency; a frequently heard statement is "since the virus has not adapted to continuing human-to-human transmission by now, it is unlikely to do so in the future." Such complacency is akin to living on a geologic fault line and failing to take precautions against earthquakes and tsunamis.

## The Source

**Figure 1:**
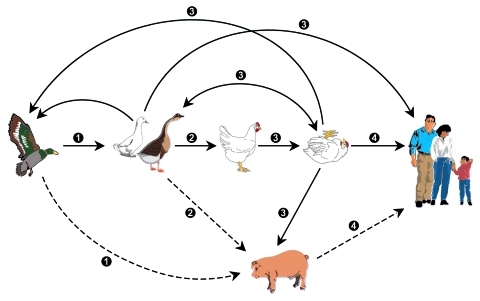
Emergence of H5N1 influenza virus and control options. A nonpathogenic H5 influenza virus is believed to have spread to domestic ducks and geese, then to domestic chickens. In chickens, the H5 virus became highly pathogenic before it was transferred back to domestic ducks and geese. The highly pathogenic H5 virus reassorted its genome with those of other influenza viruses in aquatic birds, and the resulting viruses spread to domestic poultry farms, humans, and occasionally to pigs. These viruses acquired mutations in their PB2, HA, NA, and NS genes that made them lethal to domestic and wild waterfowl and humans. Solid lines, transmission demonstrated; dotted lines, transmission postulated but not demonstrated. Multiple opportunities exist for control of highly pathogenic avian influenza: 1) prevent contact between wild and domestic poultry by use of screened poultry houses and treated water; 2) prevent contact between domestic waterfowl and gallinaceous poultry by use of screened houses and treated water and by exclusion of waterfowl from "wet markets"; 3) eradicate H5/H7 influenza viruses from gallinaceous poultry by culling or the use of vaccines that prevent disease and transmission; 4) prevent contact between poultry, pigs, and humans and make vaccines and antiviral drugs available.

Influenza A viruses are perpetuated in the wild birds of the world, predominantly in waterfowl, in which the 16 subtypes (which differ by 30% in their hemagglutinin [HA] nucleotide homology) coexist in perfect harmony with their hosts (*2**,**3*) (Figure 1). In these natural hosts, the viruses remain in evolutionary stasis, showing minimal evolution at the amino acid level over extended periods. This fact indicates that the influenza-bird association is ancient; this lack of change is surprising because influenza viruses are segmented, negative-stranded RNA viruses that have no quality-control mechanisms during replication and are highly prone to variation. After transfer to a new type of host, either avian or mammalian, influenza viruses undergo rapid evolution. However, all 16 HA subtypes, including H5 and H7, have until recently been considered to be benign in their natural hosts. This benign equilibrium between the influenza virus and its host may have changed.

## Genesis of the H5N1 Virus

Before 1997, no evidence had indicated that H5 influenza viruses could infect humans and cause fatal disease. The H7 influenza viruses were known to cause conjunctivitis in humans, and serologic studies provided evidence of subclinical human infection with the subtypes prevalent in avian live poultry markets (*4*). The precursor of the H5N1 influenza virus that spread to humans in 1997 was first detected in Guangdong, China, in 1996, when it caused a moderate number of deaths in geese and attracted very little attention (*5*). This goose virus acquired internal gene segments from influenza viruses later found in quail (A/Quail/HK/G1/97 [H9N2]) and also acquired the neuraminidase gene segment from a duck virus (A/Teal/HK/W312/97 [H6N1]) before the goose virus became widespread in live poultry markets in Hong Kong and killed 6 of 18 infected persons (*6**,**7*). This H5N1 virus was eradicated by culling all domestic poultry in Hong Kong, and the genotype has not been detected since that time. However, different reassortants continued to emerge from goose and duck reservoirs (*8*) that contained the same H5 HA glycoprotein but had various internal genes. The H5N1 viruses continued to evolve, and in late 2002, a single genotype was responsible for killing most wild, domestic, and exotic waterfowl in Hong Kong nature parks (*9**,**10*). This genotype of H5N1 spread to humans in Hong Kong in February 2002, killing 1 of 2 infected persons (*11*), and was the precursor of the Z genotype that became dominant. The Z genotype spread in an unprecedented fashion across Southeast Asia, affecting Vietnam, Thailand, Indonesia, Cambodia, Laos, Korea, Japan, China, and later Malaysia. Further analysis showed that the H5N1 influenza viruses that caused outbreaks in poultry in Japan and Korea were genetically different from those in the other countries (the V genotype) (*12**,**13*). The phylogeny of the recent Z genotype viruses showed that viruses isolated in Vietnam and Thailand formed a cluster that remained distinct from those isolated in Indonesia.

To date, >140 million domesticated birds have been killed by the virus or culled to stem its spread; as of December 2005, >130 persons have been infected in Vietnam, Thailand, Indonesia, Cambodia, and China, and 70 have died (42 in Vietnam, 14 in Thailand, 8 in Indonesia, 4 in Cambodia, and 2 in China). These recent H5N1 influenza viruses have acquired the unprecedented and disturbing capability to infect humans; to cause neurotropic disease and a high proportion of deaths in waterfowl in nature; to cause death in and be transmitted among felid species, including domestic cats (*14*); and to cause neurotropic disease and death in ferrets and mice (*15*). These incremental changes intensify concern about this H5N1 virus' pandemic potential. These traits are likely to have been acquired initially by reassortment in 2001 and 2002, when a plethora of different genotypes were detected in poultry markets and later in farms in Hong Kong (*13*). These genes were presumably acquired from viruses found in waterfowl in Southeast Asia, but the actual gene donors have not yet been identified. Since late 2002, the Z genotype has become dominant, but phylogenetically distinguishable viruses have continued to cocirculate in Indonesia and western China. These characteristics have been acquired mainly through mutations in the RNA polymerase (PB2) gene, insertions in the HA gene, and deletions in the NA and nonstructural (NS) genes. Thus, the H5N1 viruses continue to evolve, initially by reassortment and more recently by mutation and deletion (*16**,**17*). While most H5N1 influenza viruses isolated from avian species in Asia since 1997 are highly pathogenic in gallinaceous poultry, they show heterogeneous pathogenicity in other species.

In domestic ducks, the pathogenicity of the H5N1 viruses varies from high to nonpathogenic. In ferrets, most avian isolates replicate and cause respiratory tract infection, while a few strains are highly pathogenic and neurotropic (causing hind leg paralysis), and the virus has been isolated from the brain (*15*). In contrast, all isolates from humans are highly pathogenic to ferrets. A similar pattern is found in experimental infection of mice, in which most avian isolates cause respiratory infection.

## Mechanisms of Spread

Were the highly pathogenic H5N1 viruses transferred within and between countries by persons, poultry, or fomites? In previous outbreaks of highly pathogenic H5 and H7 infection in multiple countries, the spread was directly attributable to humans. The main way influenza virus is spread in poultry is by movement of poultry and poultry products; establishing good biosecurity measures on poultry farms is therefore an important defense. The poultry industry is a huge, integrated complex in Asia, and a number of firms have branches in China, Vietnam, Thailand, and Indonesia. Nonetheless, the involvement of multiple lineages of H5N1 argues against human-mediated spread from a single source. Live poultry markets are an amplifier and reservoir of infection (*18*) and probably play a role in the maintenance and spread of the virus in the region. However, a number of other factors unique to affected Asian countries make control difficult. Backyard flocks are common in the region, and these domesticated birds are not subject to any biosecurity measures. Fighting cocks are prized possessions and are often transported long distances. Fighting cocks may also play a role in the spread of infection and in transmission to humans. Many of the affected countries have a weak veterinary infrastructure and are facing highly pathogenic avian influenza outbreaks for the first time. The migrant ducks that commonly wander through rice fields scavenging fallen rice seeds are another potent mechanism for the spread of infection.

### Role of Domestic Ducks

After late 2002, when H5N1 viruses had killed waterfowl in Kowloon Park in Hong Kong, most avian H5N1 isolates isolated in Vietnam, Thailand, and Indonesia were highly pathogenic to chickens and domestic ducks. However, by late 2003 and early 2004, some avian isolates were nonpathogenic to ducks but retained their pathogenicity to chickens (*19*). Genetic analysis of these isolates showed evidence of multiple variants within single specimens (*20*). On Madin-Darby canine kidney (MDCK) cells, these viruses formed a mixture of small and large plaques that had different biologic properties. Viruses that formed large plaques were usually highly pathogenic to ducks and ferrets, whereas viruses that formed small plaques were usually nonpathogenic to both birds and ferrets. Some virus isolates formed small plaques that were pathogenic to ducks. Thus, plaque size was not a marker of pathogenicity. When ducks were orally infected with the original mixed population of H5N1 viruses, most birds died, but some excreted virus for an extended period (up to 17 days); during this time, viruses that were nonpathogenic to ducks were selected. Serologic testing of these ducks showed hemagglutination inhibition (HI) and neutralizing antibodies against the original dominant virus in the mixture; thus, immune clearance had caused the selection of the minor variants. The viruses shed on day 17 had become nonpathogenic to ducks, although they remained highly pathogenic to chickens. Sequence analysis of the HA showed that these viruses differed from the original dominant virus at multiple amino acids and were antigenically distinguishable in HI tests. Therefore, H5N1 viruses circulating in avian populations in Southeast Asia are clearly heterogeneous. Notably, this phenomenon has repeatedly been reported for other influenza viruses that are in the process of altering their interspecies transmission, including European avian H1N1 viruses that were transmitted to pigs (*21*), H9N2 viruses that were transmitted to pigs and humans, and now H5N1 viruses that are transmitted from ducks to humans. How these mixtures of codominant viruses are generated in a quasispecies is unresolved. Suggested mechanisms include mutator mutations or partial heterozygotes, but a satisfactory explanation is not available (*22*).

A subdominant population of H5N1 viruses is presumably selected in ducks after the immune response clears the dominant virus. The subdominant population appears to be uniformly nonpathogenic to ducks, as if this is the natural situation for influenza in the duck. Whether further selection will occur against the polybasic cleavage site in the HA and the pathogenicity-determining sites in PB2 and NS remains to be seen. These viruses' loss of pathogenicity to ducks, but retention of pathogenicity to chickens and presumably to humans, has been a problem associated with their eradication. In Vietnam, for example, disease signs were used as the criteria for identifying H5N1 infection in ducks. Thus, the duck has become the Trojan horse of highly pathogenic H5N1 influenza in Asia (*20*).

### Role of Migratory Birds

**Figure 2:**
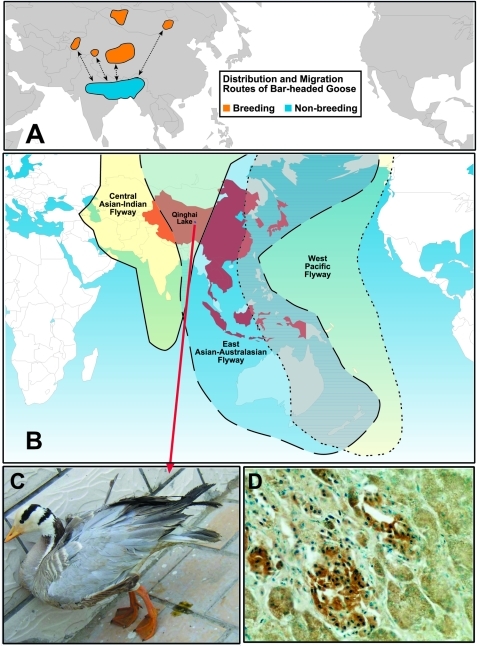
igration routes of Asian birds. A) Distribution and migration routes of bar-headed geese (courtesy of P. Leader). B) The Asia-Pacific region contains >240 species of migratory birds. The 3 flyways run primarily in a north-south direction, overlapping and extending from Australia/New Zealand to India, Central Asia, and Siberia. The outbreak of highly pathogenic (HP) H5N1 in migratory waterfowl at Qinghai Lake, China, affected primarily bar-headed geese (*Anser indicus*); however, other species, including gulls and ducks, were affected (*16**,**17*). The outbreak started in early May 2005, and by June >5,000 birds had died. The birds exhibited neurologic signs, inability to stand, diarrhea, and death. Systemic infection was detected in all organs tested. C) Bar-headed goose infected with HP H5N1 influenza virus. D) Immunostain of goose pancreas, using H5 monoclonal antibodies (magnification ×400). (C and D, courtesy of H. Chen). Countries shown in red have had outbreaks of HP H5N1 since 2004. The geographic range of H5N1 may be extended by bar-headed geese or by ducks that are less susceptible to lethal infection.

Migratory waterfowl are generally believed to be the main reservoir of all 16 subtypes of influenza A viruses, including H5 and H7 subtypes. However, less agreement is found regarding the role of migratory waterfowl in the initial spread of highly pathogenic H5N1 viruses across eastern Asia in 2003. The isolation of highly pathogenic H5N1 from herons, egrets, and peregrine falcons in Hong Kong in 2003 and 2004 leaves no doubt that wild migratory birds can be infected and may spread disease to local poultry flocks. The outbreak in Qinghai Lake (*16**,**17*) proves that these highly pathogenic H5N1 influenza viruses are transmissible among migratory waterfowl. The migration route of shorebirds in the east Asian-Australasian flyway does overlap the areas that have had H5N1 outbreaks, although the virus has been notably absent in Taiwan, Malaysia (except for occasional outbreaks near the Thai border), and western Australia (Figure 2). The role of migratory birds in the transmission and spread of highly pathogenic H5N1 viruses is still unclear. However, the recent outbreak of H5N1 infection in bar-headed geese and other species in Qinghai Lake is a cause for concern because these birds migrate southward to the Indian subcontinent, an area that has apparently not been affected by H5N1 avian influenza. If the virus were to become entrenched in India, its geographic range would be substantially extended, and the pandemic threat would increase accordingly (*17*). A mutation in the PB2 gene (residue E627K) associated with pathogenicity in mammals (*16**,**17*) has been found in viruses isolated from birds in Qinghai Lake; this finding has caused concern that this mutation will be transferred to other migratory birds (e.g., wild ducks) and will be spread because not all infected birds die.

Although culling domestic poultry to contain the spread of highly pathogenic H5N1 virus is considered an acceptable agricultural practice, culling migratory birds is not acceptable to any international authority (Food and Agriculture Organization of the United Nations [FAO], the World Organization for Animal Health [OIE], the World Health Organization [WHO]). The idea of culling migratory birds must be strongly discouraged, for it could have unknown ecologic consequences. Instead, since highly pathogenic H5N1 has been demonstrated in migratory birds, the poultry industries of the world must adapt measures such as increased biosecurity (Figure 1), the use of vaccines, or both.

Early detection and aggressive control measures allowed Japan, South Korea, and Malaysia to eradicate H5N1 virus soon after its introduction into those countries' poultry flocks, demonstrating that rapid and determined responses can keep the virus from gaining a foothold. In other countries in Asia, delayed detection and response caused the virus to become entrenched across a wide region, and eradication at this stage has become a formidable undertaking.

## Agricultural Vaccines

The need for H5N1 vaccines for domestic poultry is increasing. Adopting a policy to use vaccines in poultry is an important decision for agricultural authorities in countries such as Thailand (a major poultry exporter) and Vietnam. Both countries are investigating their specific needs. While considerable data exist on the efficacy of influenza vaccines in domestic chickens, little comparable information is available regarding ducks. The pros and cons of the use of vaccines in poultry have been reviewed (*23*). Current technologies permit discrimination between vaccinated and naturally infected birds; however, vaccines are not standardized on the basis of antigen content. "Good" and "bad" agricultural vaccines are in use.

### Good Agricultural Vaccines

Good agricultural vaccines provide protection from disease despite lack of a close antigenic match between the vaccine and circulating strain and reduce the virus load below the level of transmissibility. They do not provide sterilizing immunity: vaccinated birds may excrete low levels of virus after challenge infection. Sentinel unvaccinated birds are kept in each house to monitor for virus shedding, antigenic drift, or both.

### Bad Agricultural Vaccines

Bad agricultural vaccines prevent disease signs but do not prevent shedding of transmissible levels of virus. They also promote undetected spread of virus on farms and to live poultry markets and promote antigenic drift. China and Indonesia have adopted poultry vaccination to control H5N1, and Vietnam has begun vaccine trials in poultry. However, the resurgence of H5N1 in Indonesian poultry and pigs (*24*) and the detection of H5N1 in apparently healthy birds in live poultry markets in China (*17*) suggest that some vaccines are of suboptimal quality or that coinfection masks disease. The adoption of a vaccine strategy for H5N2 virus in Mexico in the 1980s reduced disease signs but has not eliminated the H5N2 virus from the region; instead, vaccination may have contributed to the virus' widespread presence in Central America and to its antigenic drift (*25*).

## H9N2 and Cross-protection

The clinical signs of infection with highly pathogenic H5N1 virus may be masked by cross-protection by other influenza subtypes, but this fact is often overlooked. During the initial outbreak of highly pathogenic H5N1 in Hong Kong in 1997, chickens in the live poultry markets exhibited no disease signs, yet samples from apparently healthy chickens, ducks, and quail showed highly pathogenic H5N1 in each of the poultry markets surveyed (*26*). Surveillance showed that multiple influenza subtypes were cocirculating, including 2 lineages of H9N2, the first represented by the G1 lineage (A/Quail/Hong Kong/G1/97 [H9N2]) and the other by G9 (A/Chicken/Hong Kong/G9/97 [H9N2]). The G1 lineage has the same 6 internal gene segments as the index H5N1 human isolate (A/Hong Kong/156/97 [H5N1]) and is believed to have been the donor of these genes during reassortment that produced the original H5N1 human strain in 1997 (*27*). In laboratory studies, chickens previously infected with H9N2 (A/Quail/Hong Kong/G1/97 [H9N2]) were protected from disease signs and death when challenged with highly pathogenic H5N1, but the chickens shed H5N1 virus in their feces (*28*). Further studies in inbred chickens established that the cross-protection was due to cell-mediated immunity and that it could be transferred by CD8+ T cells but not by antibodies (*29*).

The possible effect of cocirculating influenza viruses on the pathogenicity of highly pathogenic H5N1 in Vietnam, Thailand, and elsewhere in Asia has not been resolved. To date, no other subtypes of influenza A viruses have been reported in poultry in Vietnam or Thailand. Surveillance of live poultry in Hong Kong and in Nanchang (*30*) suggests that other influenza A viruses are cocirculating in live poultry markets and on duck farms. Definitive information is required to understand the ecology of influenza and the possible masking of disease signs caused by H5N1.

## Conclusion

Conventional wisdom about pandemic influenza holds that a pandemic is inevitable and that the only question remaining is "When?" The H5N1 virus continues to evolve and spread, with additional human infections occurring in Vietnam, Cambodia, Indonesia, China, and Thailand. If this virus acquires human-to-human transmissibility with its present fatality rate of 50%, the resulting pandemic would be akin to a global tsunami. If it killed those infected at even a fraction of this rate, the results would be catastrophic. While the high pathogenicity of the Qinghai bar-headed goose isolate is a continuing threat to poultry and humans, perhaps the most insidious threat comes from unobserved transmission through wild and domestic ducks. The isolation of H5N1 virus from bar-headed geese in Qinghai Lake in southern China in 2005 originated from unobserved infection in poultry markets and suggests that highly pathogenic H5N1 viruses continue to circulate unseen among poultry in China (*17*). We cannot afford simply to hope that human-to-human spread of H5N1 will not happen and that, if it does, the pathogenicity of the virus will attenuate. Notably, the precursor of the severe acute respiratory syndrome (SARS)–associated coronavirus (*31*) repeatedly crossed species barriers, probably for many years, before it finally acquired the capacity for human-to-human transmission, and its pathogenicity to humans was not attenuated. We cannot wait and allow nature to take its course. SARS was interrupted by early case detection and isolation, but influenza is transmissible early in the course of the disease and cannot be controlled by similar means. Just 1 year before the catastrophic tsunami of December 2004, Asian leaders rejected a proposed tsunami warning system for the Indian Ocean because it was too expensive and the risk was too remote. This mistake must not be repeated in relation to an H5N1 avian influenza pandemic. We must use this window of opportunity to prepare and to begin prepandemic implementation of prevention and control measures.^1^

## Footnotes

1 Since this article was written, the H5N1 influenza virus has continued to spread in migratory birds to Turkey, Croatia, and Romania. The global spread of this H5N1 in migratory birds and domestic poultry is inevitable. The question is, "When will it acquire sustained human-to-human transmission?"
